# Supplementary material for: The Change in Glucagon Following Meal Ingestion Is Associated with Glycemic Control, but Not with Incretin, in People with Diabetes
Source: J Clin Med. 2021 Jun 4;10(11):2487. doi: 10.3390/jcm10112487 (PMC8200068; doi:10.3390/jcm10112487)
Supplement: Supplementary file 1 [file jcm-10-02487-s001.zip › jcm-1185565-SI.pdf]

Journal of
Clinical Medicine

MDPI

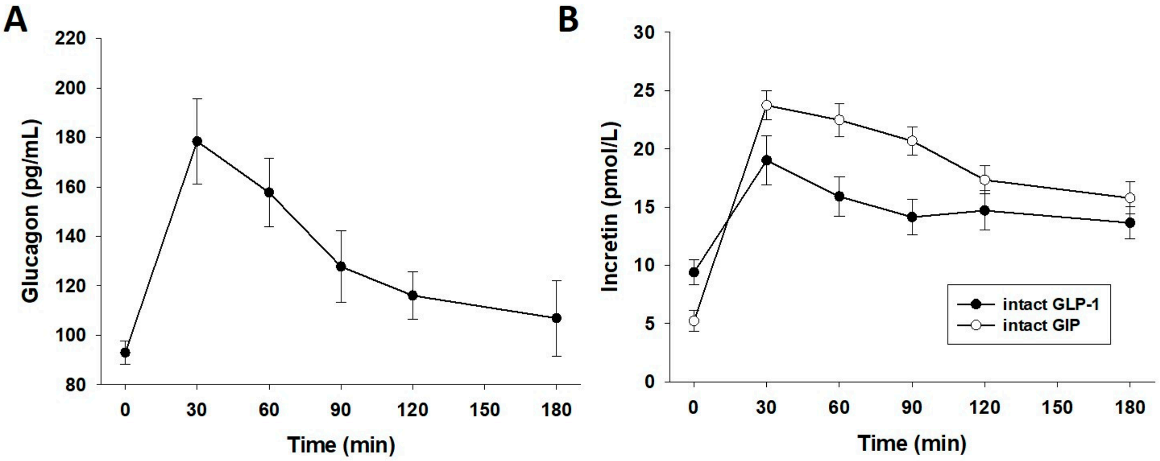

Figure S1. Changes in glucagon levels (A) and incretin levels (B) after ingestion of a standard mixed meal in diabetes people (n=36).

Table S1. Clinical and laboratory characteristics of the noninsulin user.

Variables	Value
n	217
Age, years	58.8 ± 11.6
Gender, male, *n* (%)	144 (66.4)
Body mass index, kg/cm²	26.0 ± 3.3
Waist circumference, cm	90.7± 8.3
Systolic BP, mmHg	138.1 ± 17.8
Diastolic BP, mmHg	82.6 ± 10.5
DM duration, years	7.4 ± 7.2
HbA1c, %	8.5 ± 2.2
Glucose, mg/dL	161.5 ± 57.2
C-peptide, ng/mL	2.4 ± 1.2
HOMA IR(%)	1.4 ± 1.1
ALT, U/L	34.7 ± 27.4
Creatinine, mg/dL	1.1 ± 0.2
eGFR, mL/min	69.7 ± 13.6
Antidiabetic regimen	
Sulfonylurea, *n* (%)	134 (61.8)
Metformin, *n* (%)	155 (71.4)
Thiazolidinedione, *n* (%)	17 (7.8)

α-Glucosidase inhibitor, *n* (%)	14 (6.5)

Data are expressed as mean ± standard deviation or frequencies (%).

BP, blood pressure; DM, diabetes mellitus; iGLP-1, intact glucagon-like peptide 1; iGIP, intact glucose-dependent insulinotropic polypeptide; HOMA IR, homeostasis model assessment of insulin resistance; ALT, alanine aminotransferase; eGFR, estimation of the glomerular filtration rate.

Table S2. Blood levels of glucose, C-peptide, glucagon, glucagon/insulin ratio and intact incretin before and 30 min after ingestion of a standard mixed meal in the noninsulin user (n = 217).

Variables	Fasting levels	30-min Postmeal levels	*p*
Glucose, mg/dL	163.2 ± 60.3	254.2 ± 69.2	<0.001
C-peptide, ng/mL	2.3 ± 1.0	3.6 ± 1.5	<0.001
Glucagon, pg/mL	85.6 ± 34.5	80.4 ± 47.2	0.012
Glucagon/insulin ratio	14.7 ± 12.5	6.0 ± 8.4	<0.001
iGLP-1, pmol/L	5.7 ± 3.9	11.3 ± 8.4	<0.001
iGIP, pmol/L	3.9 ± 4.1	22.4 ± 6.7	<0.001

Data are expressed as mean ± standard deviation.

p values are calculated using Wilcoxon signed-rank test.

iGLP-1, intact glucagon-like peptide 1; iGIP, intact glucose-dependent insulinotropic polypeptide.

Table S3. Correlational analysis of the relationships between Δglucagon/insulin ratio and clinical and laboratory variables in the noninsulin user (n = 217).

Variables	ΔGlucagon/insulin ratio	
	r	*p*
Age	-0.011	0.888
Body mass index, kg/cm^2	0.065	0.389
Waist circumference, cm	0.149	0.050
Systolic BP, mmHg	-0.008	0.916
Diastolic BP, mmHg	-0.106	0.159
DM duration, years	-0.135	0.073
HbA1c, %	0.218	0.003
ΔGlucose, mg/dL	0.061	0.418
ΔC-peptide, ng/mL	0.003	0.974
Log (ΔiGLP-1, pmol/L)	-0.081	0.294

ΔiGIP, pmol/L	-0.069	0.360
ALT, U/L	0.145	0.053
eGFR, mL/min	-0.002	0.981

ΔiGLP-1 was logarithmically transformed. *r* and *p* values are calculated using the Pearson correlation analysis. *r*, correlation coefficient; BP, blood pressure; DM, diabetes mellitus; iGLP-1, intact glucagon-like peptide 1; iGIP, intact glucose-dependent insulinotropic polypeptide; ALT, alanine aminotransferase; eGFR, estimation of the glomerular filtration rate

Table S4. Hierarchical multiple regression analyses predicting Δglucagon/insulin ratio in the noninsulin user (*n* = 217).

Variables	Model 1		Model 2		Model 3	
	β	*p*	*β*	*p*	*β*	*p*
Age, years	0.018	0.805	-0.022	0.777	-0.030	0.697
Gender, female	0.085	0.250	0.086	0.260	0.078	0.306
HbA1c, %	0.213	0.005	0.282	0.001	0.304	0.001
ΔGlucose, mg/dL			0.058	0.459	0.055	0.496
ΔC-peptide, ng/mL			0.171	0.048	-0.084	0.299
Log (ΔiGLP-1, pmol/L)			0.171	0.073	-0.147	0.072
ΔiGIP, pmol/L			-0.147	0.914	0.006	0.938
Use of sulfonylurea					0.143	0.079
Use of metformin					-0.030	0.697
Adjusted R^2	0.039		0.042		0.051	
F	3.404	0.019	2.076	0.049	2.016	0.041

ΔiGLP-1 was logarithmically transformed.

β, corrected regression coefficient; iGLP-1, intact glucagon-like peptide 1; iGIP, intact glucose-dependent insulinotropic polypeptide.

Table S5. Hierarchical multiple regression analyses predicting Δglucagon levels in the noninsulin user (*n* = 217).

Variables	Model 1		Model 2		Model 3	
	β	*p*	*β*	*p*	*β*	*p*
Age, years	0.076	0.231	0.042	0.515	0.042	0.542
Gender, female	0.113	0.077	0.105	0.112	0.084	0.225
HbA1c, %	0.380	<0.001	0.426	<0.001	0.406	<0.001
Fasting glucose, mg/dL	-0.001	0.995	-0.032	0.727	-0.029	0.766
ΔC-peptide, ng/mL			0.037	0.618	0.040	0.607

Log (ΔiGLP-1, pmol/L)			0.014	0.847	0.016	0.829
ΔiGIP, pmol/L			0.100	0.151	0.135	0.063
HOMA IR(%)					-0.045	0.529
Adjusted R^2	0.161		0.159		0.134	
F	11.261	<0.001	6.464	<0.001	4.692	<0.001

ΔiGLP-1 was logarithmically transformed.

β, corrected regression coefficient; iGLP-1, intact glucagon-like peptide 1; iGIP, intact glucose-dependent insulinotropic polypeptide.
